# Age, Hypertension, and Exercise Capacity are Independently Associated with Likelihood of Multi-Vessel Disease in Patients Referred for Treadmill Exercise Testing: The Intermediate-High-Workload Treadmill Score (IHWTS)

**DOI:** 10.31083/j.rcm2404108

**Published:** 2023-04-17

**Authors:** Maria C. Arciniegas Calle, Muhannad Aboud Abbasi, Husam M. Salah, Christopher G. Scott, Laura B. Garcia Bello, Amanda R. Bonikowske, Hector R. Villarraga

**Affiliations:** ^1^Department of Cardiovascular Medicine, Mayo Clinic, Rochester, MN 55905, USA; ^2^Division of Biomedical Statistics and Informatics, Mayo Clinic, Rochester, MN 55905, USA

**Keywords:** coronary artery disease, exercise testing, Intermediate-High-Workload Treadmill Score, preventive medicine

## Abstract

**Background:**

To identify factors that increase the specificity of the treadmill exercise test (TMET), and develop a novel scoring system which accounts for functional capacity to aid in determining the need for further testing.

**Methods:**

We retrospectively evaluated the electronic health records of 600 patients who had positive TMET results and follow-up stress echocardiography from 1-January-2004, through 31-December-2016. Correlations between clinical and aerobic variables and multivessel disease (MVD) were determined. Duke Treadmill Score (DTS) was calculated and compared with a novel scoring system titled the Intermediate-High-Workload Treadmill Score (IHWTS) that used variables associated with MVD.

**Results:**

In total, 124 of 600 patients (21%) had coronary catheterization, and 51 of these patients (41%) had MVD. Mean (SD) DTS was –2.10 (6.3) among patients with MVD vs –0.16 (5) among patients without MVD (*p* = 0.06). Mean (SD) functional aerobic capacity (FAC) was 76% (20%) among patients with MVD vs 90% (21%) among patients without MVD (*p *< 0.001). Mean (SD) metabolic equivalent (MET) was 7 (2) among patients with MVD vs 8 (2) among patients without MVD (*p* = 0.002). Only 6 (12%) of patients with MVD achieved 9 MET or greater on TMET. DTS less than 4 did not distinguish between patients with and without MVD (*p* = 0.67). Age, hypertension and FAC were independently associated with MVD (all *p *< 0.05).

**Conclusions:**

Our novel scoring system IHWTS utilized age, hypertension, and FAC appeared comparable to DTS to risk-stratify patients regardless of baseline symptoms. Clinical parameters such as hypertension along with exercise functional capacity should be considered when evaluating a positive TMET result in patients that achieve an intermediate-high workload >5 Metabolic Equivalents (METs).

## 1. Introduction

The treadmill exercise test (TMET) is a widely used, cost-effective diagnostic tool for identification of coronary artery disease (CAD) [1, 2]. Depending on patient clinical characteristics and symptoms, the pretest probability of stress testing varies widely. A meta-analysis of TMET reported a mean (SD) sensitivity of 68% (16%) (range, 23%–100%) and a mean (SD) specificity of 77% (17%) (range, 17%–100%) [1]. This data suggests that although CAD can be identified in asymptomatic patients, the rate of false-positive testing is high and leads to uncertainty in clinical decision-making [3, 4].

Currently, the Duke Treadmill Score (DTS) is used to establish patient prognosis and to aid in deciding whether to refer patients with findings suggestive of CAD for coronary catheterization (CC) [5]. The DTS is a composite index that provides survival estimates by considering exercise time, angina index, and ST-segment deviation in patients with chest pain [5, 6]. The score ranges from –25 to +15, with low-risk values of ≥+5, moderate risk between –10 to +4 and high risk ≥–11 respectively. Other nomograms and scoring systems that are designed to increase the diagnostic accuracy of TMET are better than DTS for predicting CAD, but they require complicated calculations and are not practical in everyday practice [7].

The American Heart Association guidelines for stress testing recommends non-invasive stress testing as a means of risk stratification and evaluation of persons with multiple risk factors as a guide for further treatment in those with positive test results and multiple CAD risk factors [8]. However, no established tool can aid clinicians in the evaluation of an asymptomatic patient with a positive treadmill test result, given that the likelihood of a false-positive result is known to be high, particularly in those with good functional capacity and can achieve a high Metabolic Equivalents (METS) on exercise stress testing [9, 10, 11]. Accordingly, our study aims to develop a simple propensity scoring system that helps identify when positive TMET results are credible or spurious (false-positive) for patients who attain an intermediate-high metabolic workload.

## 2. Methods

### 2.1 Study Population 

The Mayo Clinic Institutional Review Board approved this study, and all patients provided authorization for use of their data. This was a retrospective longitudinal study. We reviewed the electronic health records of patients who had positive TMET results and were subsequently referred for stress echocardiography (SE) (n = 600). The final cohort used for statistical analyses included those with cardiac catheterization (n = 124). A positive TMET result was defined according to the American Heart Association guidelines as ischemic changes determined with electrocardiography (ECG) and characterized as a horizontal or downsloping ST segment greater than 1 mm at 60 to 80 milliseconds after the J point in 3 consecutive beats [12]. Endpoints for TMET were fatigue limiting further exercise, leg fatigue/claudication shortness of breath per the Borg scale, cardiac symptoms included moderate to severe anginal chest pain, sustained ventricular tachycardiac, significant decline or increase in blood pressure (BP). A semiquantitative wall motion score was assigned to each segment to calculate the left ventricle (LV) wall motion score index as the average of the scores of all segments visualized. The following scoring system is used: (1) normal or hyperkinetic, (2) hypokinetic (reduced thickening), (3) akinetic (absent or negligible thickening, e.g., scar), and (4) dyskinetic (systolic thinning or stretching, e.g., aneurysm) [13]. Multivessel coronary artery disease (MVD) was defined as luminal stenosis of at least 70% in at least two major coronary arteries or in one coronary artery in addition to a 50% or greater stenosis of the left main trunk [14]. Fitness was quantified as functional aerobic capacity (FAC), a term that is interchangeable for this purpose with aerobic capacity and functional capacity. Functional aerobic capacity was calculated as achieved METs/predicted METs based on age and sex [15, 16]. All tests were performed at Mayo Clinic in Rochester, Minnesota, from 1-January-2004, through 31-December-2016, and angiography was completed within 3 months of the treadmill test.

### 2.2 Data Collection 

Clinical, TMET, SE, and CC data were collected from the electronic health records. Blood pressure, heart rate, Borg scale during each stage of the Bruce protocol, DTS, time to resolution of ischemic ST changes, total duration of the treadmill examination, metabolic equivalent (MET), and final impressions were extracted as part of the TMET data. SE data, including pre- and post-ejection fraction (EF) response, BP, heart rate, Borg scale during each stage, DTS, total duration of treadmill examination, MET, final impressions, and areas of ischemia, were also obtained. A subgroup of patients who underwent CC was analyzed to compare patients with and without multivessel disease (MVD).

### 2.3 Statistical Analysis

Data are summarized as frequency (percent) for categorical variables and mean (SD) for continuous variables. Categorical variables were compared between groups by using the Pearson χ^2^ test, and continuous variables were compared between groups by using the 2-sample *t* test. For the subgroup of patients who underwent CC (n = 124), multivariable logistic regression analyses were performed to evaluate the association between MVD and risk factors. Results are summarized with odds ratios (ORs) and associated 95% CIs. The magnitudes of the ORs from the model with the age, hypertension, and FAC groups were used to create the Intermediate-High-Workload Treadmill Score (IHWTS). Receiver operating characteristic analysis was used to correlate IHWTS and DTS with MVD, and receiver operating characteristic curves were plotted. Area under the curve is summarized to compare continuous scores. To simplify the calculation of scores, groups were created for age (<55, 55–64, and ≥65 years) and FAC (quartiles). After breaking up the scores into groups, we calculated sensitivity, specificity, positive and negative predictive values, and accuracy. Estimates of sensitivity, specificity, and accuracy were compared between scores with the McNemar test. Generalized score statistics were used to compare positive predictive value and negative predictive value. Analysis was performed with SAS version 9.4 (SAS Institute Inc, Cary, NC, USA), and a 2-sided *p* value less than 0.05 was considered significant.

## 3. Results

### 3.1 Clinical Characteristics 

Data from 600 patients were retrospectively collected from electronic health records (26 [4%] were deceased at the time of data evaluation). In total, 558 patients (93%) were white, 12 (2%) were Asian, and 6 (1%) were African American; 306 patients (51%) had 2 or more cardiovascular risk factors, such as smoking, hypertension, diabetes mellitus, and hypercholesterolemia. Individually, 90 (15%) patients had diabetes mellitus, 306 (51%) hypertension, 180 (30%) hypercholesterolemia, and 54 (9%) were smokers at the time of testing (Table 1). Table 2 highlights clinical characteristics between patients with and those without MVD.

**Table 1.** **Patient characteristics**.

Characteristics		Value (N = 600)
Age, mean (SD), years		62 (10)
Male, No. (%)		456 (76)
HTN (%)		306 (51)
HLD (%)		54 (9)
DM (%)		90 (15)
Current smoking (%)		54 (9)
Positive SE result, No. (%)		162 (27)
CC, No. (%)		124 (21)
MVD, No. (%)		51 (41)
	DTS, mean (SD)	−2.10 (6.3)
	FAC, mean (SD), %	76 (20)
	MET, mean (SD)	7 (2)
	Symptoms, No. (%)	39 (76)
No MVD, No. (%)		73 (59)
	DTS, mean (SD)	−0.16 (5)
	FAC, mean (SD), %	90 (21)
	MET, mean (SD)	8 (2)
	Symptoms, No. (%)	27 (37)

Abbreviations: HTN, Hypertension; HLD, Hyperlipidemia; DM, Diabetes Mellitus; CC, coronary catheterization; DTS, Duke Treadmill Score; FAC, functional aerobic capacity; MET, metabolic equivalent; MVD, multivessel disease; SE, stress echocardiography.

**Table 2.** **Patient characteristics with multivessel disease**.

Characteristic	MVD		
	Present (n = 51)	Absent (n = 73)	*p* value
Age, mean (SD), y	67 (10)	62 (11)	0.007
Male, No. (%)	44 (86)	49 (67)	0.02
Positive ESE result			
RWMA index at rest	1.1 (0.2)	1.1 (0.2)	0.38
RWMA index at peak stress	1.6 (0.4)	1.4 (0.4)	0.06
Hypertension, No. (%)	43 (84)	33 (45)	<0.001
Hyperlipidemia (%)	51	73	0.07
DM (%)	51	73	0.07
Current smoker (%)	60	64	0.14
DTS, mean (SD)	−2.10 (6.3)	−0.16 (5.0)	0.06
FAC, mean (SD), %	76 (20)	90 (21)	<0.001
METs, mean (SD)	7.0 (2.0)	8.0 (2.0)	<0.001

Abbreviations: DTS, Duke Treadmill Score; ESE, exercise stress echocardiography; FAC, functional aerobic capacity; MET, metabolic equivalent; MVD, multivessel disease; RWMA, regional wall motion abnormality; DM, diabetes mellitus.

### 3.2 Treadmill Exercise Test and Exercise Stress Echocardiography Data 

Indications for TMET included screening for CAD (258 patients [43%]), chest pain (114 [19%]), and dyspnea (54 [9%]). Other indications were follow-up CAD evaluation and fitness evaluation (174 [29%]). Mean (SD) 1-minute heart rate was 130 (22) beats per minute, heart rate recovery was 22 (10) beats, ECG duration was 8 (2) minutes, MET was 7 (2), and time to resolution of ischemic changes was 5 (4) minutes. One hundred seventy-two patients (29%) had positive SE result defined by regional wall motion abnormality index of 1.13 and 129 (22%) had positive SE result defined by regional wall motion abnormality index of 1.25. Additionally, 124 patients (21%) subsequently underwent CC. Of those 124 patients who had CC, 51 patients (41%) had MVD.

Mean (SD) SE duration was 9 (2) minutes, EF at rest was 62% (6%), EF post stress was 68% (10%), wall motion index at rest was 1.04 (0.15), wall motion index post stress was 1.14 (0.29), FAC was 120% (26%), MET was 10 (3), and ischemic heart rate was 139 (22) beats per minute. Most patients (76%) completed 3 or 4 phases of the Bruce protocol.

### 3.3 Cardiac Catheterization Data 

Among 124 patients who had CC, 51 (41%) had MVD. Among this subgroup, mean (SD) DTS was –2.10 (6.3) for patients with MVD and –0.16 (5.1) for patients without MVD (*p* = 0.06). Mean (SD) FAC was 76% (21%) for patients with MVD and 90% (21%) for patients without MVD (*p *< 0.001). Mean (SD) MET was 7 (2) for patients with MVD and 8 (2) for patients without MVD (*p *< 0.001). The distribution of patient characteristics of those with and without MVD who underwent CC, as well as the DTS, FAC, and MET values, are provided in Table 1. DTS less than 4, which indicates moderate risk for MVD, did not distinguish between patients with and without MVD (*p* = 0.67). Age, hypertension, and FAC were independently associated with MVD (all* p <* 0.05) (Table 3).

**Table 3.** **Variables independently associated with multivessel disease among 124 patients who had coronary catheterization composing the high-workload treadmill scoring system (n = 124)**.

${\mathrm{Variables}}^{a}$		OR (95% CI)	${\mathrm{Points}}^{b}$
Age, y			
	<55	1.0 (Reference)	0
	55–64	1.4 (0.4–4.9)	1
	≥65	2.0 (0.6–6.4)	2
Hypertension		5.5 (2.1–14.0)	5
FAC, ${\%}^{c}$			
	<81	5.3 (1.4–20.0)	4
	81–94.5	3.0 (0.8–11.7)	2
	94.6–106	0.8 (0.1–4.6)	0
	>106	1.0 (Reference)	0

Abbreviations: FAC, functional aerobic capacity; OR, odds ratio. ^a^ Variables were independently associated with multivessel disease among patients who underwent coronary catheterization. ^b^ Score <7 indicates low risk of multivessel disease; 7–8, moderate risk; 9–11, high risk. ^c^ FAC subgroups reflect values corresponding to quartiles 1–4.

### 3.4 Intermediate-High-Workload Treadmill Score 

Logistic regression was used to examine variables correlated with MVD. Variables from the univariate analysis which were not statistically significant include male gender, body mass index, angina index, negative BP difference, ST changes and symptoms, and therefore were excluded from analysis. Only age, hypertension, and FAC were independently associated with MVD (*p *< 0.05). In order to create a score that was easy to calculate, groups were created for age (<55, 55–64, 65+) and FAC (quartiles). Using these groups, ORs were calculated (Table 3). Hypertension had the strongest association with MVD (OR, 5.5). ORs were used as the basis of the score and then the association between the novel score and MVD was examined. To aid in classification of patients, groups were created to summarize risk across groups of patients on the basis of their novel scores (Table 3). According to our score, a rating <7 indicates low risk; 7–8, moderate risk; 9–11, high risk of MVD.

**Fig. 1.**
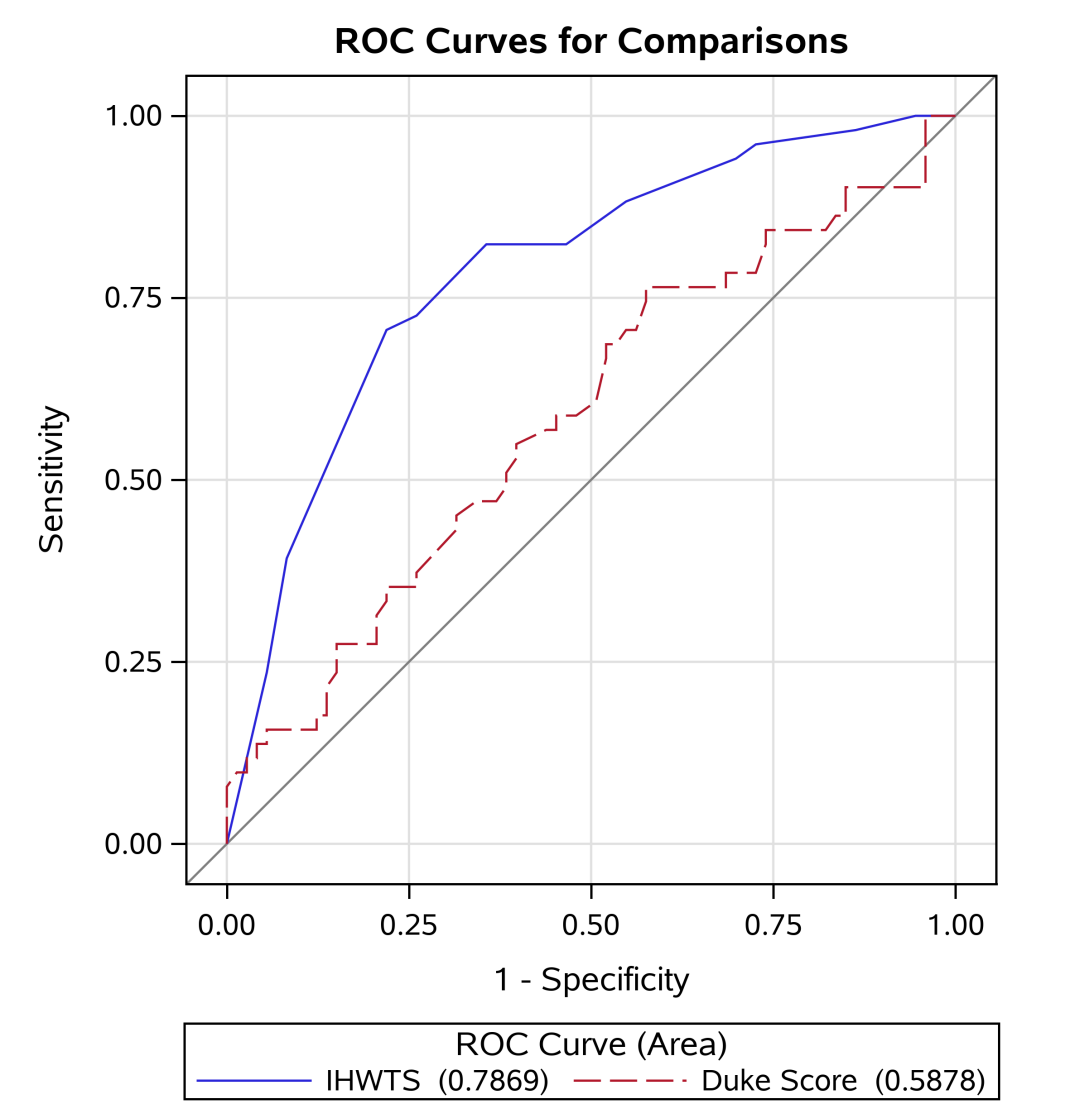
**Receiver operating characteristic curves of the Duke Treadmill Score and the High-Workload Treadmill Score**. The Intermediate-High-Workload Treadmill Score took into account age, hypertension, and functional aerobic capacity. ROC, receiver operating curves.

We compared DTS and the novel scoring system to test diagnostic accuracy among patients who had CC. Specificity, positive predictive value, negative predictive value, and accuracy were significantly greater for the novel score than DTS (Table 4, Fig. 1).

**Table 4.** **Predictive value of DTS vs IHWTS among patients who underwent coronary catheterization ${\mathrm{(n = 124)}}^{a}$**.

Value	DTS, % (95% CI)	IHWTS, % (95% CI)	*p* value
Sensitivity	84 (71–93)	82 (69–92)	0.76
Specificity	18 (10–29)	64 (52–75)	<0.001
Negative predictive value	62 (38–82)	84 (72–92)	0.04
Positive predictive value	42 (32–52)	62 (49–73)	0.001
Accuracy	45 (36–54)	72 (63–80)	<0.001

Abbreviations: DTS, Duke Treadmill Score; IHWTS, Intermediate-High-Workload Treadmill Score. ^a^ DTS and IHWTS were used to compare patients with low risk for multivessel disease vs patients with moderate or high risk for multivessel disease.

## 4. Discussion

Compared with DTS, our novel scoring system appeared to better classify patients who could attain intermediate-high workload, regardless of baseline symptoms. The novel score had significantly greater specificity, positive predictive value, negative predictive value, and accuracy compared with DTS. Our study highlights the importance of accounting for functional status and risk factors, particularly hypertension, in risk stratification and assisting in determination of a true-positive vs false-positive TMET.

The diagnostic accuracy of the TMET alone is poor, with 68% sensitivity and 77% specificity for symptomatic patients [17]. Furthermore, diagnostic accuracy varies depending on gender, age and clinical characteristics [18]. Many patients are asymptomatic and undergo the treadmill test to screen for CAD. This leads to a dilemma in clinical decision-making when an asymptomatic patient has a positive TMET result, especially when the patient can achieve an intermediate-high workload [9, 10, 11]. Fine *et al*. [11] determined that the positive predictive value of the treadmill test was 40% for a group of 7000 patients who could attain 10 MET or greater. In other words, a patient who attained greater than 10 MET and had a positive test result had a 40% probability of having CAD. Furthermore, no difference in survival was noted between patients who had positive or negative treadmill test results and had greater than 10 MET (*p* = 0.05) [11].

Current clinical practice lacks the tools needed to guide clinical decisions for patients with an intermediate to high exercise capacity and a positive TMET result. The Seattle Heart Watch Study examined cardiovascular disease risk factors, chest pain, ability to exercise for less than 6 minutes, attainment of less than 90% of predicted heart rate, and ST-segment depression to determine the importance of TMET results for risk prediction for symptomatic and asymptomatic men. TMET results were more predictive of risk for the symptomatic group [19, 20]. In addition, the Seattle Heart Watch Study did not determine a relationship between maximal or submaximal test results and predictive value of the ST-segment response in the TMET [19]. Conversely, evidence of ischemia during low workload was associated with high risk of subsequent events, such as unstable angina, myocardial infarction and death. McNeer and colleagues [21] confirmed this association and further reported that those who could perform the fourth stage of the Bruce protocol had low risk of CAD regardless of ST-segment response. These findings were recently challenged by Ermolao *et al*. [22] who found close to half of the asymptomatic athletes with equivocal or positive TMET had coronary abnormalities that warranted further testing despite a lower positive predictive value from maximal TMET. In addition, current exercise test interpretation guidelines report incidence rates of fatal and nonfatal coronary events in those who are asymptomatic yet demonstrate ST-segment depression during testing at 2.4–5.8%. Furthermore, failure to achieve target heart rate has been shown to be associated with an annual incidence rate of cardiovascular death in asymptomatic men of 1.2% to 1.7% [2, 23]. FAC as a predictor of cardiovascular events has been widely studied and shown to be an important risk factor of death due to cardiovascular and all causes. Higher physical fitness is associated with reduced all-cause mortality specifically through reduced rates of cardiovascular disease and cancer. This finding remained after adjustment for age, smoking, cholesterol level, systolic BP, fasting blood glucose level, and parental history of CAD [24]. Compared to those with higher exercise capacities, patients with reduced exercise capacity generally have less-favorable outcomes than those with good or excellent exercise capacity [25]. In addition, if a patient can achieve at least 10 METs on TMET outcomes are favorable despite any ST-segment depression or MVD independent of sex [24]. This was true in our group of patients as those who could attain a higher workload had lower incidence of MVD. A study published in 1975 hinted at this association, even before DTS was validated, and reported that patients had maximal stress with ST abnormalities if the onset of a 2-mm ST-segment depression happened at or before 3 minutes; these patients had significantly worse prognosis compared with patients who had first depression at 5 minutes. In addition, when ST depression first manifested at 7 minutes, which was near peak capacity, the incidence of coronary events was slightly greater than those with a negative test result [26]. A recent meta-analysis including 34 studies and 3352 patients that a positive exercise stress test was more helpful in younger patients (Likelihood Ratios +=4.74) than in older patients (Likelihood Ratios +=2.8) [18]. Furthermore, a cross sectional analysis examining the appropriateness of TMET request (n = 191) patients found that in patients with low pretest probably, the presence of hypertension, diabetes and dyslipidemia were more frequent in the appropriate than inappropriate indications (71%, 19% and 29% vs 43%, 8% and 16%, respectively) [27].

Other nomograms and scores have been developed with the aim of improving diagnostic accuracy of TMET and DTS, and many reportedly have better prognostic accuracy. For example, the Lauer score was better than DTS at predicting death due to all causes among patients with findings suggestive of CAD and normal ECG results [28]. The Morise, Detrano, and Veteran Affairs scores are probability scores calculated with multiple logistic regression analysis, and all of these scores diagnose CAD more accurately than the standard ST-segment response criteria utilized in the DTS. However, these scores are complicated to calculate and are not replicable in all populations. Another study determined that these other scores more accurately diagnosed CAD [7]. Our novel scoring system also had better diagnostic accuracy than DTS. It is simple to calculate and can evaluate patients with positive TMET result and good workload. This patient population represents a clinical challenge. Furthermore, we believe our study is congruent with previous literature that suggest that clinical judgement and evaluating clinical comorbidities should be taken into consideration when interpreting results of TMET.

## 5. Limitations

This study was limited by its retrospective nature. DTS was developed as a prognostic score for symptomatic patients, and thus head-to-head comparison was limited by differences from the population in which DTS was established. Larger prospective studies needed for further validation.

## 6. Conclusions 

Clinical parameters, such as comorbid conditions particularly arterial hypertension and FAC, should be considered when evaluating a patient with a positive TMET result. Our simple-to-use novel scoring system fills a gap in patient stratification by identifying those who may have MVD but are not identified by DTS, thereby increasing the specificity of TMET. To our knowledge, this is the first scoring system that aids clinicians by stratifying risk for MVD among patients with positive TMET result and intermediate-high workload.

## Data Availability

The datasets generated and/or analyzed during the current study are not publicly available due to institutional protocol and reasons, but are available from the corresponding author on reasonable request.

## Abbreviations

CAD, coronary artery disease; CC, coronary catheterization; DTS, Duke Treadmill Score; ECG, electrocardiography; EF, ejection fraction; TMET, Treadmill exercise test; FAC, functional aerobic capacity; MET, metabolic equivalent; MVD, multivessel disease; SE, stress echocardiography; ESE, exercise stress echocardiography; RWMA, regional wall motion abnormality; HTN, Hypertension; HLD, Hyperlipidemia; DM, Diabetes Mellitus.
